# Ectoparasitic mites exert non-consumptive effects on the larvae of a fruit fly host

**DOI:** 10.1017/S0031182023000744

**Published:** 2023-09

**Authors:** Collin J. Horn, Sarah Robinson, Holly Tang, Lien T. Luong

**Affiliations:** Department of Biological Sciences, University of Alberta, 11455 Saskatchewan Drive Edmonton, AB, Canada

**Keywords:** Behavioural immunity, *Drosophila*, life stages, *Macrocheles*, trait-mediated effect

## Abstract

The mere presence of predators or parasites can negatively impact the fitness of prey or hosts. Exposure to predators during an organism's development can have deleterious effects on juvenile survival and the subsequent adult stage. Currently, it is unknown if parasites have analogous impacts on host larval stages and whether these effects carry over into other subsequent life stages. However, parasites may be exerting widespread yet underestimated non-consumptive effects (NCEs). We tested if *Drosophila nigrospiracula* larvae avoid pupating near mite cues (caged *Macrocheles subbadius*) in arena experiments, and measured the rate of pupation in arenas with mites and arenas without mites. Larvae disproportionately pupated on the side of arenas that lacked mite cues. Furthermore, fewer larvae successfully pupated in arenas containing mites cues compared to arenas without mite cues. We found that ectoparasitic mites exert NCEs on *Drosophila* larvae, even though the larval stage is not susceptible to infection. We discuss these results in the context of parasite impacts on host population growth in an infectious world.

## Graphical abstract

**Figure:**
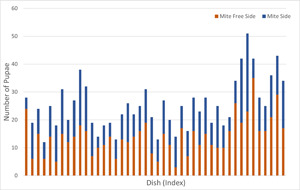

## Introduction

Natural selection has driven the evolution of parasite-avoidance behaviours and other anti-parasite defences that reduce the risk of infection and/or limit post-infection proliferation. For example, hosts can reduce infection risk by avoiding infective stages, grooming or altering their habitat use (Hart, 1994; Lefevre *et al*., 2012; Buck *et al*., 2018; Koprivnikar *et al*., 2021). The mere presence of parasites or parasite cues, *sans* infection, can influence host behaviour (e.g. changes in habitat use, auto-grooming, etc.) and induce physiological stress. In predator-prey ecology, these indirect ‘non-consumptive effects’ (NCEs) can have consequences for prey growth, survival and reproduction (Werner and Peacor, 2003; Preisser *et al*., 2005). By acting on the entire population, NCEs can have greater cumulative impacts than consumption *per se* on prey populations (Mouritsen and Poulin, 2005; Clinchy *et al*., 2013).

Recent work has extended concepts developed for predator-prey systems to disease ecology and parasite-host associations (Raffel *et al*., 2008; Rohr *et al*., 2009; Daversa *et al*., 2021). Previous reluctance to do so assumed that since parasitic infections do not cause immediate lethality, anti-parasite responses should be relatively weak. However, increasing evidence suggests otherwise (Rohr *et al*., 2009; Buck *et al*., 2018). For example, small mammals are quicker to abandon resource patches that are heavily infested with ticks compared to sites with lower infection risk (Fritzsche and Allan, 2012). Likewise, female fruit flies avoid ovipositing in habitats laden with ectoparasitic mites (Mierzejewski *et al*., 2019). Parasite avoidance may be costly, especially if it negatively affects foraging and reproductive activities (Preisser *et al*., 2005; Ferrari *et al*., 2009; Benoit *et al*., 2020). Pea aphid populations declined by 50% in the presence of parasitoid wasps even though the aphids were not a susceptible host, because wasp presence induced escape behaviours and reduced feeding opportunities (Fill *et al*., 2012). *Drosophila nigrospiracula* adults in proximity to ectoparasitic mites experienced an energetic cost of parasite exposure, driven by a combination of physiological stress and induction of energetically expensive behavioural responses, and consequently suffered reduced survival and fecundity even though they were never in direct contact with the mites (Luong *et al*., 2017*a*; Horn and Luong, 2018; Benoit *et al*., 2020).

In predator-prey systems NCEs can carry over from one developmental stage to another, and potentially across generations. Fly larvae can sense and avoid predators, and larval stages of *D. melanogaster* exposed to spiders have lower adult masses, potentially mediated by accelerated development (Krams *et al*., 2016). Furthermore, exposure to predator cues reduced the pupation success rate (i.e. survival to adulthood) of dragonfly larvae (McCauley *et al*., 2011). There is currently no evidence that parasites have NCEs on larvae, and if this impacts larval survival and/or carry over to the adult stage (Ellrich *et al*., 2016; Krams *et al*., 2016). However, larvae could benefit from sensing and responding to parasites both by (1) directly avoiding infection and (2) pupating in less infectious environments, but these responses may be costly, requiring time and energy.

Here we test the hypothesis that exposure to parasites has effects on larval behaviour (mite-cue avoidance) and development (measured as pupation success). *Drosophila* larvae possess an olfactory system and can learn olfactory cues (Python and Stocker, 2002; Scherer *et al*., 2003). Hence, it is reasonable to assume that fruit fly larvae have the potential to detect cues from ectoparasitic mites without direct contact. We tested our hypotheses using the cactiphilic fly *D. nigrospiracula* and a natural hemolymph-feeding ectoparasite *Macrocheles subbadius* (caged to avoid contact). We predict that (1) *D. nigrospiracula* larvae will preferentially pupate away from mites in a choice-test and (2) exhibit a higher rate of pupation in the total absence of mites. We also consider if exposure during the larval stage carries over into the adult stage in the form of reduced body size.

## Materials and methods

### Study system

*D. nigrospiracula* and *M. subbadius* cultures were founded from wild-caught organisms from necrotic *Carnegiae gigantea* in the Sonoran Desert (Arizona, USA). Cultures are fully described in Horn and Luong (2021).

### Mite attachment on larval stages

This experiment was designed to test whether mites attach to larval *D. nigrospiracula*, which have 3 instar stages (1–3). Larvae were transferred from a culture bottle to a 50 mL cup of 20% sucrose. After 20 min, larvae were transferred from the surface of the solution *via* a sterilized spoon to a Petri dish lined with wet filter paper. Individual 1st/2nd (*N* = 50) or 3rd (*N* = 40) instar larvae (L1/L2s and L3s respectively) were placed into a ventilated 0.5 mL Eppendorf microtube lined with moist filter paper. Three mites previously starved for 3–4 days were then added to the tube. The position of mites was observed every 30 min for 2 hours (the majority of *M. subbadius* that attach to adult flies do so within 1–2 hours, Luong *et al*., 2017*b*). The number of mites on/adjacent larvae was modelled with observation time (since experiment start) and larval stage (L3 or L1/L2) using a glm (family = Poisson). Attachment was deemed successful if a mite attached and remained attached for an additional hour. Larvae survival was checked an additional 2 hours after the exposure period.

### Pupation site and success

**Figure 1.**
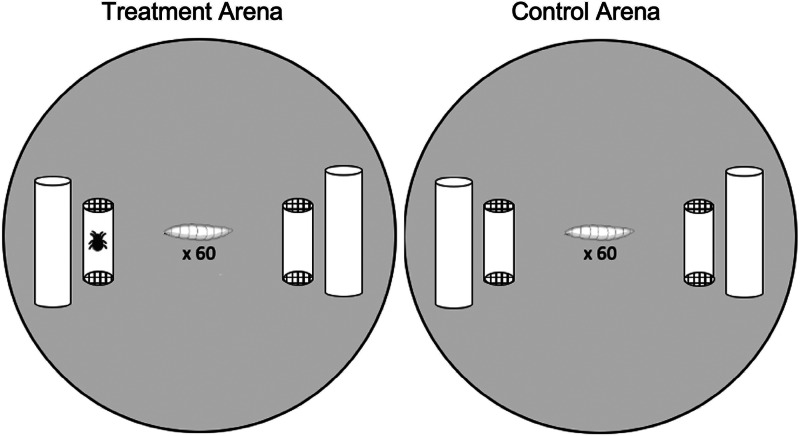
Illustration of the experimental set up. Arenas (60 mm Petri dishes) contained 2 mite cages (cropped translucent pipette tips closed with mesh) as well as *Drosophila* medium and dental rolls as a pupation substrate. Treatment arenas (left) had a side with mites in the cage and a mite-free side, the position of the cages with and without mites was alternated between dishes. Control arenas (right) had 2 empty mite cages.

This experiment tested whether larvae avoided pupating near mites, and if mite exposure, *sans* contact, had knock-on effects on larval pupation success. Pupation arenas consisted of a 60 mm aerated Petri dishes lined with *Drosophila* media and ~1 g autoclaved cactus (Fig. 1). Cotton dental rolls placed on opposite sides of the Petri dish provided pupation substrates. A mite cage (cropped translucent, yellow pipet tip with mesh ends, ~2 cm long × 0.5 cm diameter) was placed adjacent to each dental roll. Mite cages allowed mite cues to exit the cage but prevented mites from contacting the larvae. Treatment arenas (*n* = 45) had an empty cage on one side and a cage containing 5 mites on the opposite side. In control arenas (*n* = 9) both cages were empty (i.e. mite-free). Arenas were replicated over a 4-day period; for a given day sixty 3rd instar fly larvae were taken from a single culture bottle and randomly added to the centre of each arena. Petri dish arenas were sealed with parafilm and placed in an incubator (25°C, 70% relative humidity, 12 h:12 h light:dark cycle) for 5 days after which we recorded the number of pupae present, and if they occurred on the mite-side or the non-mite side in the treatment groups, after which mite cages were removed.

The proportion of pupae on the mite-side of dishes with mites (pupae on mite side/total number of pupae) was calculated for each dish, then a 1 group *t*-test (H_0_: *u* = 0.5) was used to test if larvae disproportionately pupated on the mite-free or mite-containing side. A generalized linear model (family = beta distribution, betareg function, betareg package) was used to test if the overall pupation rate was a significant predictor of the proportion of pupation on the mite side. A glm (glm.nb function, family = negative binomial, link = log, MASS package) was used to test if mite exposure affected the number of *D. nigrospiracula* pupae between dishes with and without mites.

Long term storage of arenas inevitably led to mould – in both treatment and control groups – causing adult emergence to be inconsistent. Thus, we did not record the proportion of flies that eclosed as adults. However, arenas were monitored, and adults were collected and immediately frozen so that they could be weighed (Mettler Toledo XPE105 balance, OH USA); the date of emergence but not the specific arena each fly came from was recorded. The body masses of flies from the treatment arenas were compared to flies from control arenas. Mixed effect models (lmer function) were used to test if larval mite exposure, fly sex and the exposure-sex interaction predicted the adult mass of flies, along with the random effect collection date.

## Results

### Mite attachment on larval stages

The majority of fly larvae (including 100% of L3 larvae) exposed to mites were alive 2 hours after the exposure period; 18 1st/2nd instar larvae died towards the middle and later part of this period for reasons unrelated to mites (desiccation). At the outset, mites were more likely to be on or attached to (we could not confirm if mites had attached without disturbing the assay) L1/L2s than L3s (df = 5, Δdeviance = 7.37, *P* = 0.007, 95% CI: −1.73 to −0.26). However, mites were much less likely to be observed on a larva (L1/L2 and L3) the longer the trial continued (df = 5, Δdeviance = 15.82, *P* < 0.001, 95% CI: −0.034 to −0.011). The interaction between stage and time was not a significant predictor of if mites were near larvae (df = 4, Δdeviance = 1.51, *P* = 0.22, 95% CI: −0.009 to 0.040). Although some mites initially approached the fly larvae, none remained on or attached to the larva for more than 30 min, suggesting the initial approach was likely exploratory behaviour. We did not find evidence that mites infect larvae at ecologically relevant rates or time spans.

### Pupation site and success

**Figure 2.**
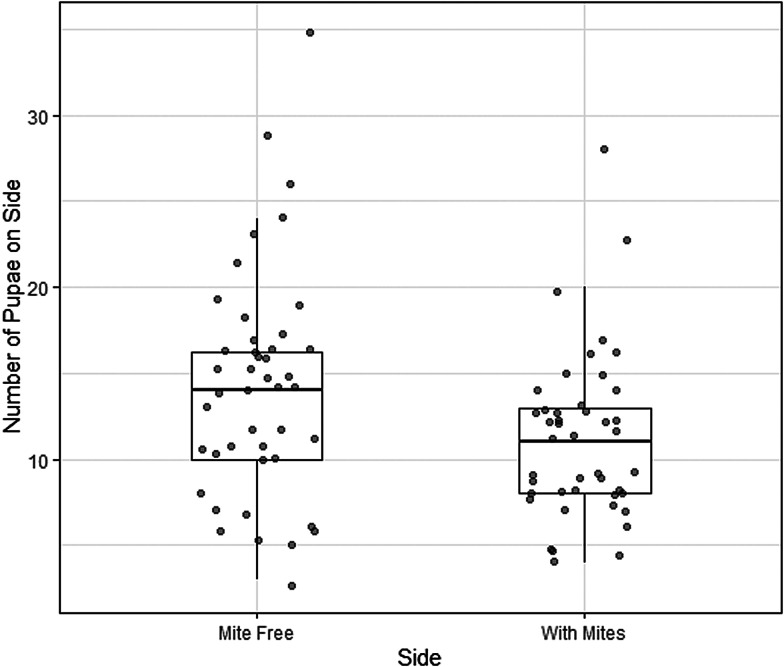
Number of larvae of the cactiphilic fly *Drosphila nigrospiracula* that pupated on the mite-free side vs the side of the arena with caged mites. Each arena contained sixty 3rd instar larvae able to move freely throughout the arena. Upper and lower box edges represent the first and third quartiles, while the middle line indicates the median.

In total, 44 of 45 of the treatment dishes (containing mite cages) were successful (1 dish was discarded due to mould). Larvae were disproportionately more likely to pupate on the side of the dish without mites (t = −2.3, df = 43, *P* = 0.028). The mean proportion of pupae on the side with mites was 0.45 (95% confidence interval [CI]: 0.41–0.49). Thus, larvae had a moderate, but statistically significant, preference for pupating away from mites (Fig. 2).

**Figure 3.**
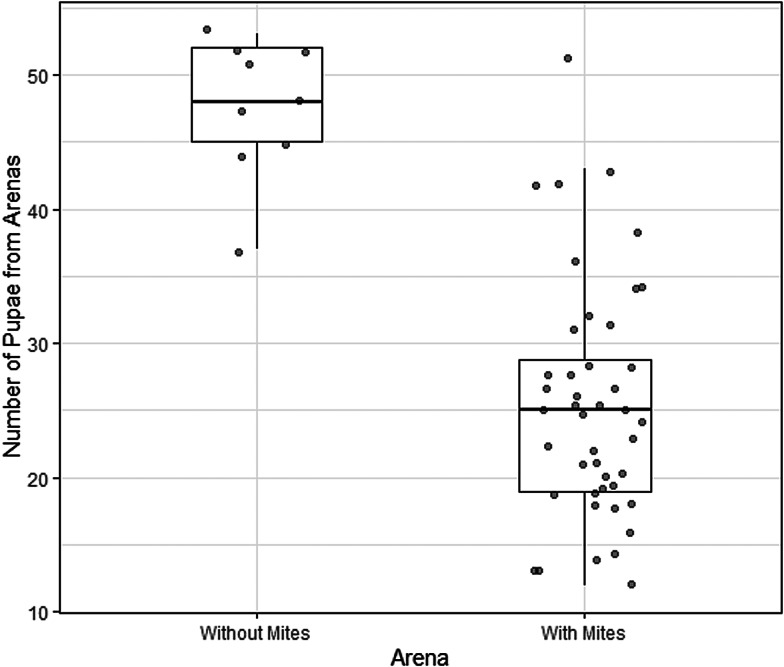
Number of *Drosphila nigrospiracula* that successfully formed pupae in treatment arenas containing caged mites (*n* = 44) or control arenas with no mites (*n* = 9). 60 larvae were placed in mite-free or mite-containing Petri dish arenas. Upper and lower box edges represent the first and third quartiles, while the middle line indicates the median.

The rate of pupation also differed between dishes containing mites (treatment arenas) and those that did not contain mites (control arenas). In the dishes containing mites there were 25.4 ± 1.3 (mean ± standard error of the mean) pupae, whereas in mite-free dishes there were 47.7 ± 1.7 pupae. Mite presence in the arena was a statistically significant predictor of the number of pupae (df = 51, Δdeviance = 40.1, *P* < 0.001, 95% CI of the regression coefficient: 0.43–0.83) (Fig. 3).

Adult flies were harvested the day of eclosion following mite exposure (or control unexposed flies) to assess differences in mass as a result of exposure (i.e. parasitic NCEs) as larvae. A total of 93 flies (56 male and 37 females) were retrieved on 5 collection dates. A male-biased sex ratio was expected from previous literature (Polak and Markow, 1995). There was no significant interaction between mite exposure and fly sex on mass (df = 89 [from lmertest], F = 0.072, P = 0.79, 95% CI: −0.22 to 0.21). Fly sex was a highly significant predictor of mass, aligning with previously observed sexual dimorphism (df = 89, F = 100.1, *P* < 0.0001, 95% CI: 0.42–0.62). Mite exposure as larvae was only a marginal predictor of adult mass (df = 51, F = 3.1, *P* = 0.087, 95% CI: −0.25 to 0.013). Unexposed females were on average 2.43 ± 0.06 mg, and unexposed males were on average 1.90 ± 0.04 mg. Among flies exposed to mites as larvae, female flies were on average 2.24 ± 0.06 mg and male flies were 1.75 ± 0.04 mg.

## Discussion

We tested for NCEs of ectoparasitic mites on fly larvae. Even though mites do not attach to the larval stage, we observed avoidance behaviours and reduced pupation success. We found that larvae responded to the presence of mites and preferentially pupated on the mite-free side of the treatment arenas. Additionally, mites exerted NCEs on the larvae by reducing pupation success.

*Drosophila* larvae can actively avoid predators and parasitoids (Ebrahim *et al*., 2015; Krams *et al*., 2016); our results extend this observation to ectoparasites. The ability to avoid mites may be directly beneficial to larvae, but our observations indicate that *M. subbadius* do not attach to the L3 larvae and largely ignored L1/L2 larvae during the observation period. It's not clear if the larval stages are resistant to infection and/or the mites actively avoid attaching to larvae because of the low potential for dispersal. Female mites (usually gravid) attach to adults flies for nutrients and dispersal between ephemeral habitats. Most likely, larvae benefit in the long-term by pupating in less infectious environments because mite infection is highly deleterious to the survival and reproductive success of adult flies (Polak, 1996). This may be particularly important for newly eclosed adults which are not fully sclerotized and cannot immediately take flight to escape mites – wings must unfurl and dry before flight. Remarkably, the presence of mites elicited avoidance behaviour (pupation site selection) by the larval stage even though it is not susceptible to infection by the mites.

Furthermore, the pupation success (i.e. survival during metamorphosis) was adversely affected even though mites do not infect larvae or cause direct larval mortality. It is not clear how larvae sensed mites, however the visual system of larvae is simpler than that of adult flies (Keene and Sprecher, 2012). Fly larvae may be able to sense mites by olfactory/chemical cues, and/or vibrations when mites contact the mesh barrier. The artificial nature of the Petri dishes may have confined or concentrated the cues, which would otherwise be more diffuse in the wild. However, the flies and mites typically interact within small pockets of necrotic tissue, so the size of the observational arena is biologically relevant. The presence of mites may negatively affect feeding among the larvae with knock-on effects on pupation success. Pea aphid exposed to parasitoids exhibited increased escape behaviours and consequently reduced feeding (Fill *et al*., 2012). Future research is needed to examine the NCEs of parasite exposure on host foraging behaviour in the fly-mite system. When dragonfly larvae were exposed to predator cues, pupation success rate but not adult body size was reduced (McCauley *et al*., 2011). Similarly, we only detected a marginal effect of parasite exposure on adult body size. This may be because food was provided *ad libitum*, which may have mitigated the deleterious effects of parasite exposure on the surviving larvae/pupae. Host responses to parasites may also trigger a stress response in larvae that is not necessarily adaptive. Stress responses may cause a cascade of deleterious physiological responses that potentially increase the risk of mortality (i.e. failure to pupate), as has been seen in predator-prey relationships (Preisser, 2009; Sheriff *et al*., 2009; McCauley *et al*., 2011). Such stress-induced NCEs could have significant implications for the ecology of fear and host-parasite dynamics.

Computer models of fly-mite populations found that the earlier the impacts of NCEs are in the fly lifespan, the larger the impacts of mites are on host population growth (Horn *et al*., 2022). Theoretical work suggests that, if natural enemies are not the only source of mortality, attacks earlier in the lifespan will have larger impacts on the population growth of the prey/host species (Godfray and Waage, 1991; Murdoch and Briggs, 1996). However, until now there was no evidence parasite NCEs affected host development and survival to adulthood. Our results show that larval exposure to parasites impacts pupation site selection and induces carryover effects (metamorphosis). Thus, previous studies (Horn *et al*., 2022) may have underestimated the non-consumptive impacts of mites on the growth rate of fly populations by only accounting for impacts of mites on adult flies.

Parasitologists and ecologists increasingly recognize that parasites have diverse ecological effects beyond infection. NCEs are a major avenue by which parasites may exert influence outside of infection. This study provides evidence that an ectoparasite has NCEs on the behaviour and survival of host larvae to the pupal stage. Impacts of natural enemies on pre-reproductive stages can have outsized impacts on host population growth. Investigating the NCEs of parasites on larval stages may reveal widespread yet underestimated impacts of parasites.

## Data Availability

Raw data and R code can be accessed online (OSF doi: 10.17605/OSF.IO/CVU28).
